# Extracorporeal Membrane Oxygenation-Related Nosocomial Infection after Cardiac Surgery in Adult Patients

**DOI:** 10.21470/1678-9741-2020-0068

**Published:** 2021

**Authors:** Jing Wang, Liangshan Wang, Ming Jia, Zhongtao Du, Xiaotong Hou

**Affiliations:** 1 Management Office of Nosocomial Infection, Beijing Anzhen Hospital, Capital Medical University, Beijing, People’s Republic of China.; 2 Center for Cardiac Intensive Care, Beijing Anzhen Hospital, Capital Medical University, Beijing, People’s Republic of China.

**Keywords:** Cross Infection, Extracorporeal Membrane Oxygenation, Odds Ratio, Incidence, Risk Factors, Body Mass Index, Hospital Mortality, Cardiac Surgical Procedures

## Abstract

**Introduction:**

The evaluation of extracorporeal membrane oxygenation-related nosocomial infection (ECMO-related NI) in a homogeneous cohort remains scarce. This study analyzed ECMO-related NI in adult patients who have undergone cardiac surgery.

**Methods:**

From January 2012 to December 2017, 322 adult patients who have received ECMO support after cardiac surgery were divided into the infection group (n=131) and the non-infection group (n=191). ECMO-related NI was evaluated according to demographic data, surgical procedures, and ECMO parameters.

**Results:**

The incidence of ECMO-related NI was 85.4 cases per 1000 ECMO days. *Acinetobacter baumannii* was the most common pathogen causing blood stream infection and respiratory tract infection. Prolonged duration of surgery (*P*=0.042) and cardiopulmonary bypass assist (*P*=0.044) increased the risk of ECMO-related NI. Body mass index (odds ratio [OR]: 1.077; 95% confidence interval [CI]: 1.004-1.156; *P*=0.039) and duration of ECMO support (OR: 1.006; 95% CI: 1.003-1.009; *P*=0.0001) were the independent risk factors for ECMO-related NI. Duration of ECMO support > 144 hours (OR: 2.460; 95% CI: 1.155-7.238; *P*<0.0001) and ECMO-related NI (OR: 3.726; 95% CI: 1.274-10.895; *P*=0.016) increased significantly the risk of in-hospital death.

**Conclusion:**

Prolonged duration of ECMO support was an independent risk factor for NI. Surgical correcting latent causes of cardiopulmonary failure and shortening duration of ECMO whenever possible would reduce susceptibility to NI.

**Table:** 

Abbreviations, acronyms & symbols				
BMI	= Body mass index		ICU	= Intensive care unit
BSI	= Blood stream infection		LVEF	= Left ventricular ejection fraction
CABG	= Coronary artery bypass grafting		NI	= Nosocomial infection
CI	= Confidence interval		NYHA	= New York Heart Association
CPB	= Cardiopulmonary bypass		OR	= Odds ratio
CRRT	= Continuous renal replacement therapy		RTI	= Respiratory tract infection
ECMO	= Extracorporeal membrane oxygenation		SWI	= Sternal wound infection
ECMO-related NI	= Extracorporeal membrane oxygenation-related nosocomial infection		UTI	= Urinary tract infection
ELSO	= Extracorporeal Life Support Organization		VA	= Venoarterial

## INTRODUCTION

Extracorporeal membrane oxygenation (ECMO) is one of the most important life support techniques for patients with severe but reversible cardiac and/or pulmonary failure. Despite advance in technique and management of ECMO, nosocomial infection (NI) is still one of severe complications of ECMO that remarkably increases morbidity and mortality^[1]^.

Comparing with other patients in intensive care unit (ICU), the patients undergoing ECMO are with increasing possibility of NI due to cannulation disrupting the skin barrier and allowing pathogens invasion and the hollow fiber structure of the oxygenator contributing to pathogens establishment^[2,3]^. Several studies have analyzed the risk factors and pathogens of ECMO-related NI^[4-6]^, but different studies have obvious heterogeneity from baseline characteristics including populations (pediatric *vs.* adult patients), number of patients, concomitant diseases, causes of cardiac or pulmonary failure, ECMO cannulation access (percutaneous *vs.* intrathoracic), and ECMO support mode (venoarterial [VA] *vs.* venovenous). Such heterogeneity may affect the incidence, risk factors, and pathogens of ECMO-related NI.

In addition, the data on ECMO-related NI in adult patients after cardiac surgery remain scarce.

Therefore, we focused on ECMO and cardiac surgery parameters and investigated a single-center large sample homogenous group to analyze the incidence, risk factors, and pathogens of ECMO-related NI in adult patients who have undergone cardiac surgery and VA-ECMO support.

## METHODS

### Patient Population

From January 2012 to December 2017, a total of 35,327 adult patients underwent cardiac surgery at the Beijing Anzhen Hospital, Capital Medical University, People’s Republic of China. Among those patients, 463 suffering from severe but reversible cardiac and/or pulmonary failure received ECMO support. This study’s inclusion criteria were: (1) age ≥ 18 years; (2) intraoperative or postoperative ECMO support; (3) no infection identified by specimen culture (blood, sputum, urine, or wound secretion, etc.) before ECMO support; (4) VA-ECMO support ≥ 48 hours.

According to the inclusion criteria, 322 patients were recruited to this study. Baseline characteristics, surgical parameters, ECMO parameters, and in-hospital outcomes were reviewed from medical records. This is a retrospective cohort study in accordance with the Ethical Guideline of the Committee on Human Experimentation of our institution, and the informed consent was obtained from the patients.

### ECMO Procedures

The indications for ECMO were identified by extracorporeal life support physicians and cardiac surgeons. VA-ECMO mode with cannulation via femoral artery and vein was a predominant procedure duo in all patients who underwent cardiac surgery. Systemic anticoagulation was achieved by continuous intravenous heparin infusion to maintain activated clotting time in a range of 180-200 s. ECMO flow was initially set with a rate of 100 ml/kg/min and the flow level was tailored to achieve adequate tissue perfusion evaluated by lactate level and end-organ function. The management protocols of ECMO followed the Extracorporeal Life Support Organization (ELSO) guidelines^[7]^.

### Respiratory Management During ECMO

Lung-protective ventilatory strategies were employed during ECMO support. The parameters of mechanical ventilation included: tidal volume 4-6 ml/kg (ideal body weight), respiratory rate < 12/min, inhaled oxygen concentration 30-40%, positive end-expiratory pressure 6-10 cmH_2_O, and airway platform pressure < 25 cmH_2_O. The protocols of extubation included: (1) sober mind, good limb movement, strong airway self-cleaning ability; (2) hemodynamic stability, normal central venous pressure; (3) adequate tissue perfusion, serum lactate concentration < 3 mmol/L, urine > 1 ml/(kg*h) under sufficient blood volume or continuous renal replacement therapy; (4) PaO_2_/FiO_2_ > 200 mmHg without pulmonary edema; (5) no predictable secondary thoracotomy.

### Definition of ECMO-Related NI

NI occurring 24h after initiation and 48h after discontinuation of ECMO was defined as ECMO-related NI^[8]^. The type of ECMO-related NI included blood stream infection (BSI), respiratory tract infection (RTI), urinary tract infection (UTI), and sternal wound infection (SWI). The diagnosis and definition of ECMO-related NI were based on the Centers for Disease Control and Prevention definitions for NI^[9]^. Isolation of pathogenic microorganisms was related to clinical symptoms, typical inflammation characteristics in blood samples, and radiographic findings.

### Anti-Infective Therapy

Patients received antibiotic prophylaxis with second-generation cephalosporins at initiation of ECMO support. If ECMO-related NI occurred, antibiotic was adjusted according to the culture results. The conventional dose of antibiotic was started and regulated according to renal function and drug concentration monitoring.

### Statistical Analysis

Statistical analysis was performed using an extensively admissive software program, the SAS software (SAS Institute Inc 2013. SAS/ACCESS® 9.4 Interface to ADABAS: Reference. Cary, NC: SAS Institute Inc.). Continuous data and categorical data were expressed as mean ± standard deviation and percentage, respectively. Variance test was used to address nonpaired samples for comparing normally distributed parameters, and Wilcoxon rank sum test was used for comparing nonparametric variables. The qualitative variables were compared using chi-squared test or Fisher’s exact probability test. The multiple logistic regression analysis was conducted to identify the risk factors of ECMO-related NI, BSI, and in-hospital death. P<0.05 was considered to be statistically significant.

## RESULTS

### Baseline Characteristics and In-Hospital Outcomes of ECMO Support

The total ECMO support time in 322 patients was 1675.34 days. The average age of this cohort was 57.19±11.85 years, and male patients accounted for 74.22% (n=239). Patients in the infection group underwent longer duration surgeries (447.92±206.07 min *vs*. 402.35±191.35 min, *P*=0.042) and received higher percentage of cardiopulmonary bypass (CPB) assist (58.02% *vs*. 46.60%, *P*=0.044) than those in the non-infection group. Detailed baseline characteristics were showed in Table 1.

**Table 1:** Baseline characteristics of ECMO populations.

Variables	All patients (n=322)	Infection group (n=131)	Non-infection group (n=191)	*P*-value
Age (year)	57.19±11.85	57.02±12.35	57.30±11.52	0.959
Male (%)	239 (74.22)	98 (74.81)	141 (73.82)	0.897
BMI	24.35±3.57	24.81±3.55	24.04±3.55	0.126
Hypertension	156 (48.45)	66 (50.38)	90 (47.12)	0.573
Diabetes mellitus	72 (22.36)	32 (24.43)	40 (20.94)	0.497
Chronic pulmonary disease	8 (2.48)	2 (1.53)	6 (3.14)	0.480
NYHA class				
I or II	206 (63.98)	79 (60.30)	127 (66.49)	0.256
III or IV	116 (36.02)	52 (39.70)	64 (33.51)	
LVEF	49.35±8.41	48.76±9.06	49.75±8.26	0.311
Timing of operation				
Scheduled operation	285 (88.51)	117 (89.31)	168 (87.96)	0.859
Emergency operation	37 (11.49)	14 (10.69)	23 (12.04)	
Type of operation				
CABG	254 (78.88)	97 (74.05)	157 (82.20)	0.078
Cardiac valves surgery	58 (18.01)	28 (21.37)	30 (15.71)	
Other cardiac surgeries	10 (3.11)	6 (4.58)	4 (2.09)	
Reoperation	22 (6.83)	11 (8.40)	11 (5.76)	0.376
Duration of operation (min)	420.78±200.20	447.92±206.07	402.35±191.35	0.042
Use of CPB	165 (51.24%)	76 (58.02%)	89 (46.60%)	0.044

BMI=body mass index; CABG=coronary artery bypass grafting; CPB=cardiopulmonary bypass; ECMO=extracorporeal membrane oxygenation; LVEF=left ventricular ejection fraction; NYHA=New York Heart Association

Comparing with the non-infection group, the patients in the infection group underwent longer ECMO support (153.53±105.33 hours *vs*. 105.21±71.79 hours, *P*<0.001) with lower ECMO weaning rate (56.49% *vs*. 70.16%, *P*=0.012); details were presented in Table 2. Regarding major complications and outcomes, acute renal failure requiring continuous renal replacement therapy (42.75% *vs*. 31.41%, *P*=0.037 ) was more common and survival rate to discharge was lower (41.98% *vs*. 53.92%, *P*<0.001) in the infection group than in the non-infection group (Table 3).

**Table 2:** Patients’ ECMO-related parameters and outcomes.

Variables	Infection group (n=131)	Non-infection group (n=191)	*P*-value
Indications for ECMO support			
Failure to wean off CPB	21 (16.03)	36 (18.85)	0.541
Refractory cardiac-pulmonary failure	98 (74.81)	137 (71.73)	
Cardiac arrest	12 (9.16)	18 (9.42)	
ECMO implantation site			
ICU or ward	59 (45.04)	80 (41.88)	0.647
Operation room	72 (54.96)	111 (58.12)	
ECMO cannula			
Femoral puncture	34 (25.95)	43 (22.51)	0.718
Femoral incision	87 (66.41)	135 (70.68)	
Transthoracic cannula	10 (7.64)	13 (6.81)	
Duration of ECMO (hours)	153.53±105.33	105.21±71.79	<0.001
ECMO weaning success	74 (56.49)	134 (70.16)	0.012

CPB=cardiopulmonary bypass; ECMO=extracorporeal membrane oxygenation; ICU=intensive care unit

**Table 3:** Patients’ major complications and in-hospital outcomes.

Variables	Infection group (n=131)	Non-infection group (n=191)	*P*-value
Stroke			
Ischemic	2 (1.52)	4 (2.09)	0.798
Hemorrhagic	5 (3.82)	5 (2.62)	
Major bleeding	27 (20.61)	32 (16.75)	0.379
CRRT	56 (42.75)	60 (31.41)	0.037
Limb ischemia requiring surgery	4 (3.05)	5 (2.62)	0.996
Outcome of unweaning off ECMO			
Death	57 (43.51)	55 (28.80)	0.012
Cardiac transplantation	0 (0)	2 (1.04)	
Survival to discharge	55 (41.98)	103 (53.92)	< 0.001

CRRT=continuous renal replacement therapy; ECMO=extracorporeal membrane oxygenation

### Incidence and Pathogenic Microorganisms of NI

**Fig. 1:**
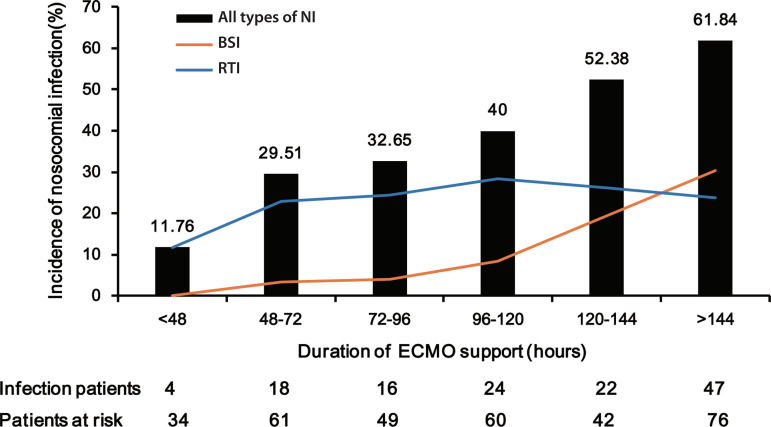
Incidence of nosocomial infection (NI) according to the duration of extracorporeal membrane oxygenation (ECMO) support. BSI=blood stream infection; RTI=respiratory tract infection

**Fig. 2:**
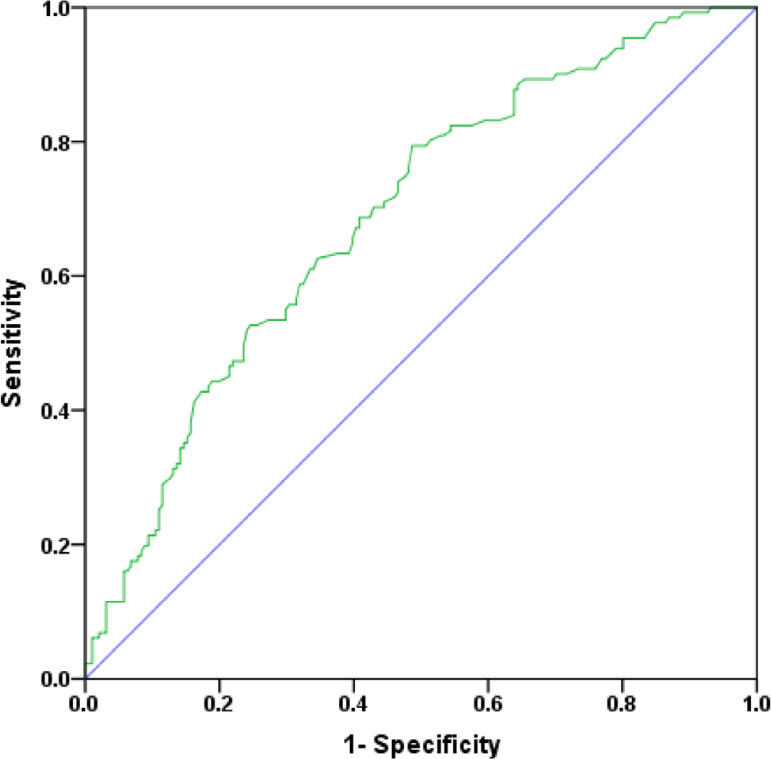
Receiver operation characteristic curve for predicting nosocomial infection with duration of extracorporeal membrane oxygenation support (area under the curve 0.689, 95% confidence interval 0.631-0.747, P<0.0001).

Among 322 patients, 131 suffered from a total of 143 infectious events. These included 40 cases of BSI, 76 cases of RTI, 10 cases of UTI, and 17 cases of SWI. The incidence of ECMO-related NI was 85.4 cases per 1000 ECMO days. The incidences of ECMO-related NI and BSI were gradually increased with prolonging duration of ECMO support (Figure 1). The receiver operating characteristic curve showed that a cutoff value was 92 hours (sensitivity 0.794, specificity 0.497), which indicated a significantly increased risk of NI with ECMO support > 92 hours (Figure 2).

Gram-negative bacteria were predominant pathogens and accounted for 64.01% of the total culture-positive pathogens, Gram-positive bacteria for 27.75%, and fungi for 8.24%. *Acinetobacter baumannii* was the most common pathogen of BSI and RTI. *Candida* (70%) was the main fungal pathogen. The pathogenic microorganisms of NI are showed in Table 4.

**Table 4:** Microorganism species causing ECMO-related NI.

Microorganism species	Samples [n (%)]				All patients (n=131)
	Blood	Tracheal secretion	Wound secretion	Urine	
Gram-negative pathogens	119 (51.07)	106 (45.49)	6 (2.58)	2 (0.86)	233
Acinetobacter baumannii	36 (45.57)	41 (51.90)	2 (2.53)	0 (0)	79
Pseudomonas aeruginosa	15 (46.88)	17 (53.12)	0 (0)	0 (0)	32
Klebsiella pneumoniae	18 (60.00)	11 (36.67)	1 (3.33)	0 (0)	30
Enterobacter cloacae	16 (84.21)	0 (0)	1 (5.26)	2 (10.53)	19
Others	34 (46.58)	37 (50.68)	2 (2.74)	0 (0)	73
Gram-positive pathogens	45 (44.55)	50 (49.50)	5 (4.95)	1 (0.99)	101
Alpha streptococcus	8 (53.33)	6 (40.00)	1 (6.67)	0 (0)	15
Micrococcus	5 (33.33)	8 (53.33)	1 (6.67)	1 (6.67)	15
Micrococcus catarrhalis	6 (40.00)	9 (60.00)	0 (0)	0 (0)	15
Staphylococcus epidermidis	6 (54.55)	3 (27.27)	2 (18.18)	0 (0)	11
Others	20 (44.44)	24 (53.33)	1 (2.22)	0 (0)	45
Fungi	30 (100)	0 (0)	0 (0)	0 (0)	30
Candida albicans	21 (100)	0 (0)	0 (0)	0 (0)	21
Candida tropicalis	4 (100)	0 (0)	0 (0)	0 (0)	4
Others	5 (100)	0 (0)	0 (0)	0 (0)	5

ECMO-related NI=extracorporeal membrane oxygenation-related nosocomial infection

### Multivariate Analysis of Risk Factors for NI and In-Hospital Death

**Fig. 3:**
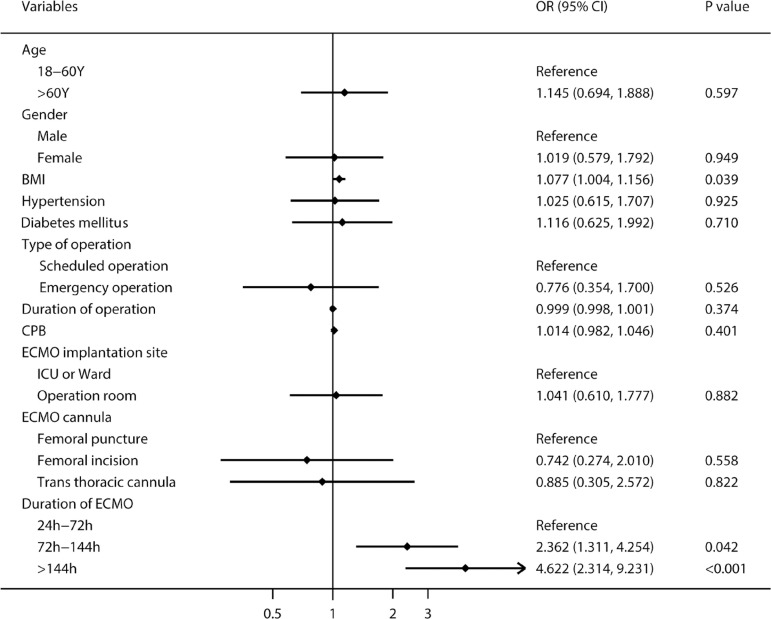
The multivariate logistic regression analysis of risk factors for extracorporeal membrane oxygenation (ECMO)-related nosocomial infection. BMI=body mass index; CI=confidence interval; CPB=cardiopulmonary bypass; ICU=intensive care unit; OR=odds ratio

**Fig. 4:**
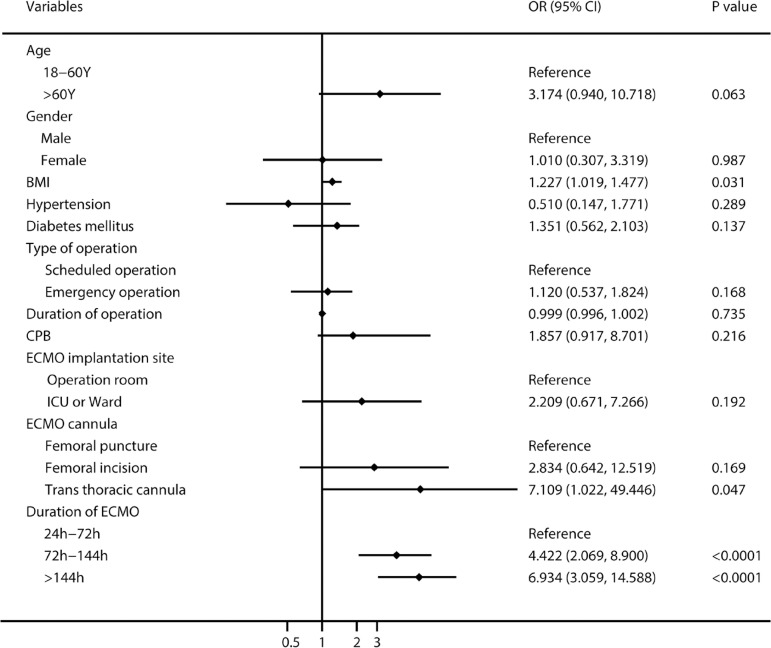
The multivariate logistic regression analysis of risk factors for extracorporeal membrane oxygenation (ECMO)-related blood stream infection. BMI=body mass index; CI=confidence interval; CPB=cardiopulmonary bypass; ICU=intensive care unit; OR=odds ratio

**Fig. 5:**
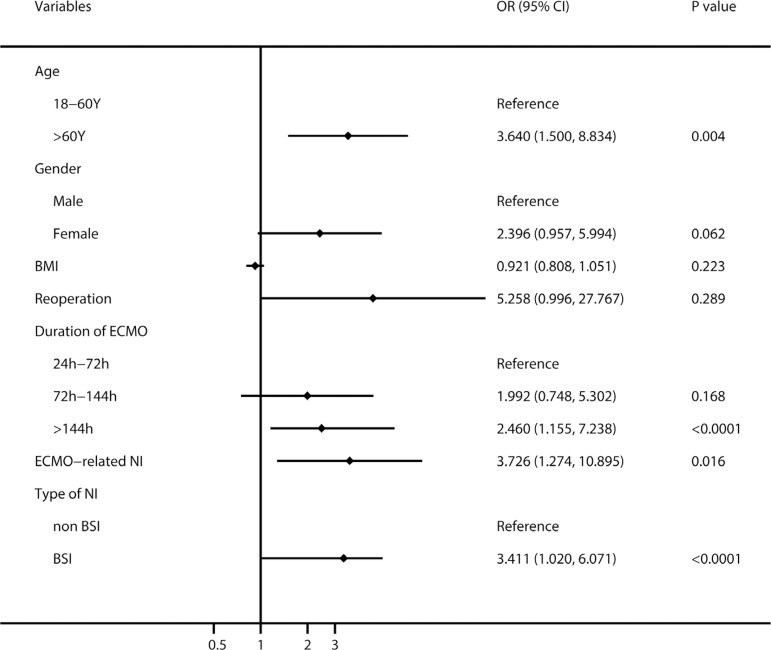
The multivariate logistic regression analysis of risk factors for in-hospital death. BMI=body mass index; BSI=blood stream infection; CI=confidence interval; ECMO=extracorporeal membrane oxygenation; NI=nosocomial infection; OR=odds ratio

Multivariate logistic regression analysis (Figure 3) revealed that body mass index (BMI) (adjusted odds ratio [OR]: 1.077; 95% confidence interval [CI]: 1.004-1.156; *P*=0.039) and duration of ECMO support (OR: 1.006; 95% CI: 1.003-1.009; *P*=0.0001) were independent risk factors for ECMO-related NI, and the risk of NI with 72-144 hours and > 144 hours of ECMO support increased by 2.4 and 4.6 times than that of < 72 hours of ECMO support, respectively. In terms of risk factors for BSI, Figure 4 showed that increased BMI (OR: 1.227; 95% CI: 1.019-1.477; *P*=0.031), transthoracic cannula (OR: 7.109; 95% CI: 1.022-49.446; *P*=0.047), and ECMO support > 72 hours increased significantly the risk of BSI. For in-hospital death, Figure 5 revealed that age > 60 years, ECMO support > 144 hours, ECMO-related NI, and BSI increased significantly the risk of in-hospital death.

## DISCUSSION

To the best of our knowledge, this investigation was the largest single-center observational study analyzing characteristics of ECMO-related NI in adult patients who underwent cardiac surgery. In this study, 40.68% (131/322) of the patients suffered from NI during ECMO support with a NI incidence of 85.4 cases/1000 ECMO days. However, the latest literature reported that the rate of NI was 30.4% during ECMO support^[7]^, and the incidence of ECMO-related NI ranged from 11.92 to 43.3 cases/1000 ECMO days^[8,10]^. The ECMO population was often companied by severe comorbidities, patients in our study were exposed to invasive procedures and suffered from severe surgical complications before ECMO support, which may increase the risk of ECMO-related NI. Moreover, the patients often required prolonging time of mechanical ventilation, central venous catheters, and urinary catheter, or there was an additional needing for intra-aortic balloon pump during ECMO support or continuous renal replacement therapy due to deleterious hemodynamic conditions after cardiac surgery, which also contributed to high incidence of ECMO-related NI in our study.

We evaluated causes of NI and found that prolonged duration of ECMO support was an independent risk factor for ECMO-related NI. Our result was in line with several previous studies^[11,12]^. And our results also showed that BMI was an independent risk factor for ECMO-related NI. Some studies^[13,14]^ suggested that obesity was an independent risk factor for NI. The precise mechanism of increasing risk of NI with increasing BMI remains unknown, but obesity is closely associated with impaired immune responses^[15]^, which may disturb the body’s defense against infections.

Moreover, prolonged surgical time and CPB assist during surgery increased the risk of ECMO-related NI. Prolonged surgical time increased open chest duration, which increased the possibility of pathogens invasion. And CPB caused ischemia-reperfusion injury of lung and induced systemic inflammatory response, which may disturb immune response, and then increase susceptibility to infections^[16,17]^. In addition, prolonged surgical time and CPB assist remarkably increased risk of multiple organ failure. And multiple organ failure was an independent risk factor for infections^[18]^. However, the multivariate regression analysis did show that prolonged surgical time and CPB assist were not independent risk factors for ECMO-related NI. Patients who have undergone prolonged surgical time and CPB assist were more susceptible to suffer from severe multiple organ failure, and then lost chance of ECMO support or death occurred within 48 hours of ECMO support, which might explain the abovementioned difference in our results.

Regarding the type of ECMO-related NI, BSI was the most common in most studies, followed by RTI, UTI, and SWI^[19,20]^. But our study showed that RTI was predominant, followed by BSI, SWI, and UTI. Most patients were assisted by invasive mechanical ventilation before ECMO support and required prolonging mechanical ventilation due to severe heart failure, which may explain the difference of infection types between our ECMO cohort and the general ECMO cohort. Moreover, antibiotic prophylaxis may affect infection types. Despite of lacking study-demonstrated benefits, antibiotic prophylaxis is commonly used at ECMO centers, according to surveys of ELSO member sites^[21,22]^.

The pathogens species causing ECMO-related NI change with therapy chronology and antibiotics renewing. ELSO reported that the majority of culture-positive pathogens in patients who have undergone ECMO were coagulase-negative *Staphylococci, Candida albicans*, and *Pseudomonas aeruginosa*^[23]^. In our study, the Gram-negative bacilli were the most common pathogens, which were often isolated species in the ICU settings. And the most common pathogen causing BSI and RTI was *Acinetobacter baumannii*, which was conditioned pathogen. Severe morbidities after cardiac surgery might disturb the immune system and break ecological balance of the conditioned pathogen and the host, resulting in infections related to conditioned pathogen. In addition, *Candida* was the main fungal pathogen in both BSI and RTI, which was associated with prolonged use of wide-spectrum antibiotics during ECMO support.

### Limitations

This study had limitations regarding susceptibility to inherent bias from retrospective data from a single center. But a homogeneous cohort and relatively large number of patients allowed us to draw strong conclusions.

## CONCLUSION

The prolonged duration of ECMO support was an independent risk factor for NI after cardiac surgery. Therefore, the best strategies to prevent ECMO-related NI in these patients were as follows: timely surgery correcting causes of cardiopulmonary failure - such as myocardial ischemia due to susceptible acute occlusion of grafts, unsatisfactory repair of heart valves, and delayed cardiac tamponade to shortening duration of ECMO support - and considering to wean mechanical ventilation whenever possible, when patients meet protocols of extubation.

**Table:** 

Authors' roles & responsibilities	
JW	Substantial contributions to the conception or design of the work; or the acquisition, analysis, or interpretation of data for the work; drafting the work or revising it critically for important intellectual content; final approval of the version to be published
LW	Acquisition, analysis, or interpretation of data for the work; final approval of the version to be published
MJ	Acquisition, analysis, or interpretation of data for the work; final approval of the version to be published
ZD	Acquisition of data for the work; final approval of the version to be published
XH	Interpretation of data for the work; drafting the work or revising it critically for important intellectual content; final approval of the version to be published
